# The SBT6.1 subtilase processes the GOLVEN1 peptide controlling cell elongation

**DOI:** 10.1093/jxb/erw241

**Published:** 2016-06-16

**Authors:** Sarieh Ghorbani, Kurt Hoogewijs, Tamara Pečenková, Ana Fernandez, Annelies Inzé, Dominique Eeckhout, Dorota Kawa, Geert De Jaeger, Tom Beeckman, Annemieke Madder, Frank Van Breusegem, Pierre Hilson

**Affiliations:** ^1^Department of Plant Systems Biology, VIB, B-9052 Ghent, Belgium; ^2^Department of Plant Biotechnology and Bioinformatics, Ghent University, B-9052 Ghent, Belgium; ^3^Department of Organic Chemistry, Ghent University, B-9000 Ghent, Belgium; ^4^Institut Jean-Pierre Bourgin, UMR1318 INRA-AgroParisTech, Saclay Plant Science, F-78026 Versailles, France

**Keywords:** Arabidopsis, hypocotyl elongation, peptide processing, protease inhibitor, serpin, signaling peptides, subtilase.

## Abstract

The *GOLVEN* (*GLV*) gene family encode small secreted peptides involved in important plant developmental programs. Little is known about the factors required for the production of the mature bioactive GLV peptides. Through a genetic suppressor screen in *Arabidopsis thaliana*, two related subtilase genes, *AtSBT6.1* and *AtSBT6.2*, were identified that are necessary for GLV1 activity. Root and hypocotyl *GLV1* overexpression phenotypes were suppressed by mutations in either of the subtilase genes. Synthetic GLV-derived peptides were cleaved *in vitro* by the affinity-purified SBT6.1 catalytic enzyme, confirming that the GLV1 precursor is a direct subtilase substrate, and the elimination of the *in vitro* subtilase recognition sites through alanine substitution suppressed the *GLV1* gain-of-function phenotype *in vivo*. Furthermore, the protease inhibitor Serpin1 bound to SBT6.1 and inhibited the cleavage of GLV1 precursors by the protease. *GLV1* and its homolog *GLV2* were expressed in the outer cell layers of the hypocotyl, preferentially in regions of rapid cell elongation. In agreement with the SBT6 role in GLV precursor processing, both null mutants for *sbt6.1* and *sbt6.2* and the *Serpin1* overexpression plants had shorter hypocotyls. The biosynthesis of the GLV signaling peptides required subtilase activity and might be regulated by specific protease inhibitors. The data fit with a model in which the GLV1 signaling pathway participates in the regulation of hypocotyl cell elongation, is controlled by SBT6 subtilases, and is modulated locally by the Serpin1 protease inhibitor.

## Highlight

Maturation of GLV signaling peptides requires two SBT6 subtilases. SBT6 proteolytic activity is further regulated by the Serpin1 inhibitor, implying a complex network that controls cell elongation in Arabidopsis.

## Introduction

Phytohormones are generally considered to be the major players in plant intercellular signaling. However, secreted peptides are now also recognized as important molecules in cell-to-cell communication because of their involvement in key developmental processes, such as meristem maintenance, organ abscission, cell elongation, cell proliferation and differentiation, gravitropism, and defense (Stahl and Simon, 2012; Ghorbani *et al.*, 2014). In the complete genome sequence of *Arabidopsis thaliana*, more than 1000 genes have been found that encode putative secreted peptides with a potential signaling function (Lease and Walker, 2006; Ohyama *et al.*, 2008; Ghorbani *et al.*, 2015; Tavormina *et al.*, 2015), but, thus far, the molecular mechanisms that control production and perception of these peptides have been studied only for a few of these genes.

Recently, a novel family of genes has been identified that encode small secretory peptides, designated either GOLVEN (GLV), root meristem growth factors (RGF), or CLAVATA/embryo surrounding region-like (CLEL). For clarity, hereafter, these peptides will be referred to according to their GLV nomenclature (Fernandez *et al.*, 2013*a*). The family consists of 11 members that are expressed during different developmental stages and in diverse plant tissues. Particular members show highly specific transcription patterns, usually restricted to a few cell types only (Fernandez *et al.*, 2013*b*). Some are involved in the regulation of root meristem maintenance (Matsuzaki *et al.*, 2010), auxin carrier turnover during gravitropic responses (Whitford *et al.*, 2012), root hair formation, and lateral root development (Meng *et al.*, 2012; Fernandez *et al.*, 2013*b*, 2015). Specifically, the GLV1 signal modulates auxin gradients in Arabidopsis hypocotyls. Up- or down-regulation of the *GLV1* gene hampers the lateral redistribution of auxin upon gravistimulation of the hypocotyl and inhibits its gravitropic response (Whitford *et al.*, 2012).

Peptides secreted by multicellular eukaryotes are generally synthesized as larger precursor proteins that are biologically inactive and undergo several proteolytic steps, including removal of the signal peptide sequence and subsequent cleavage. In plants, only two enzymes have been shown to process preproproteins into mature signaling peptides. In fact, only a few natural plant protease substrates have been described until now (Tsiatsiani *et al.*, 2012). Finally, additional post-translational modifications are often required to achieve full biological functionality (Matsubayashi, 2014).

In the case of the GLV family, the predicted proteins consist of a central variable region that links two conserved domains: an N-terminal domain coding for a signal peptide that targets the precursor to the secretory pathway, probably cleaved off by signal peptide peptidases, and a C-terminal domain, designated the GLV motif, which codes for the bioactive mature peptide. For example, the mature bioactive GLV1 peptide contains a 14-amino-acid sequence derived from the 86-amino-acid precursor (Whitford *et al.*, 2012). Thus, proteolytic processing steps are needed to remove portions of the precursor polypeptides, leading to the secretion of mature GLV peptides.

GLV proproteins carry sites in their variable region that may be targeted by subtilisin-like serine proteases, also known as subtilases, which cleave peptide bonds at or near di-basic residues (Schaller *et al.*, 2012). When compared with other eukaryotic taxons, most subtilase subgroups are under-represented in plants, whereas those related to pyrolysin have expanded up to 56 members in Arabidopsis, suggesting that these proteases may have evolved with novel target repertoires (Rautengarten *et al.*, 2005). As some subtilases have already been shown to process secreted peptides, they could be involved in the production of GLV signals (Liu *et al.*, 2007; Srivastava *et al.*, 2008, 2009). For example, the Arabidopsis SUBTILASE1.1 (SBT1.1) is required for the processing of the PHYTOSULFOKINE4 propeptide (Srivastava *et al.*, 2008), SBT6.1 is involved in the maturation of the RAPID ALKALINIZATION FACTOR23 (RALF23) (Srivastava *et al.*, 2009), and SBT3.5 regulates the processing of PECTIN METHYLESTERASE17, which modulates the esterification level of pectins on the cell wall (Sénéchal *et al.*, 2014).

As some of the Arabidopsis subtilases might be implicated in GLV protein maturation, their possible requirement for bioactive GLV peptide production was investigated. A genetic suppressor screen identified two subtilases that are necessary for the GLV1 signal activity and that participate in the control of organ growth by modulating cell expansion. The subtilase action on the GLV signaling pathway was confirmed by the characterization of the subtilase biochemical activity on GLV precursors.

## Materials and methods

### Growth conditions

Unless otherwise specified, *Arabidopis thaliana* (L.) Heynh., accession Columbia-0 (Col-0) seeds were surface-sterilized and sown on half-strength Murashige and Skoog (½MS) medium (Duchefa Biochemie B.V.) suplemented with 1% (w/v) agarose and 1.5% (w/v) sucrose at pH 5.8, and stratified for at least 2 days at 4 °C. Seedlings were germinated in illuminated growth chambers under a 16h light/8h dark cycle (100 µmol m^−2^ s^−1^) at 21 °C. For root growth analysis, plates were slanted at a 45 ° angle with respect to the gravity vector for 7 days. For hypocotyl length measurements, seeds were surface-sterilized, stratified at 4 °C for at least 2 days in liquid ½MS media, exposed to the light for 6h, and then transferred to darkness for 2–5 days under continuous rotation. Imaged root and hypocotyl features were measured with the ImageJ software (http://rsbweb.nih.gov/ij/).

### Recombinant DNA constructs and Arabidopsis lines

The *GLV1*-overexpressing (*GLV1* ^*OE*^), *GLV2* ^*OE*^, *GLV1pro:GUS-GFP*, *GLV1pro:NLS-GFP-GFP*, *GLV2pro:GUS-GFP* and *GLV2pro:NLS-GFP-GFP* lines have been described previously (Fernandez *et al.*, 2013*b*). The subtilase mutant collection was a gift from Dr Thomas Altmann (Institut für Biochemie und Biologie, Genetik, Universität Potsdam, Golm, Germany). The presence of T-DNA inserts in the Arabidopsis *SBT* genes was confirmed by polymerase chain reaction (PCR) analysis with gene-specific primers and a left border T-DNA primer (for all primer sequences, see Supplementary Table S1 at *JXB* online). The *GLV1* ^*OE*^ *sbt* mutant lines were produced by introducing the *35S:GLV1* construct (carrying either the kanamycin or the phosphinothricin resistance gene) into the *sbt* mutant lines via floral dip (Clough and Bent, 1998). At least five independent *GLV1* gain-of-function lines were analysed per transformed mutant lines. Plant DNA was isolated and analysed by PCR. The double *sbt6.1-1 sbt-6.2* knockout mutant lines, with or without the *GLV1* ^*OE*^ transgene, were obtained through crosses and genotyped at the F2 generation (for primer sequences, see Supplementary Table S1).

To generate *GLV1* ^*OE*^ and *Serpin1* ^*OE*^ cassettes, the full-length coding sequences of the genes were ampliﬁed by PCR from ﬁrst-strand cDNA of Arabidopsis with gene-speciﬁc primers extended with either the *att*B1 or *att*B2 sites for Gateway recombinational cloning. The resulting PCR fragments were captured by BP clonase reaction in an entry clone derived from pDONR221. Overexpression constructs were obtained by LR recombination between the entry clones and the destination vector pK7GW2 or pB7GW2 (Karimi *et al.*, 2002). PCR reactions were run with High Fidelity Platinum Taq DNA Polymerase (Invitrogen).

For *Serpin1pro:GUS-GFP*, the promoter sequence (approximately 1500bp upstream of the start codon) was amplified by PCR from the Arabidopsis Col-0 genomic DNA with Gateway-compatible primers (Supplementary Table S1). The promoter amplicon was cloned into pBGWFS7 (Karimi *et al.*, 2002), generating pBGWFS7PAtSRP1, which codes for a transcriptional fusion with a *GFP:GUS* translational fusion gene. The bimolecular fluorescence complementation (BiFC) expression clones (p35S:ORF:nGFP and p35S:ORF:cGFP) were generated in the pK7m34GW destination vector (http://www.psb.ugent.be/gateway/index.php) (Boruc *et al.*, 2010). In all cases, the enhanced green fluorescent protein (EGFP) fragments were fused at the C end of the tested interactors.

To generate *GLV1* ^*OE*^ lines that carried mutations in either of their subtilase recognition motifs, primers were designed to replace four and five amino acids with alanine at the first and second subtilase recognition motifs, respectively (Supplementary Table S1). A Gateway-compatible *GLV1* ^*OE*^ cassette carrying a mutation in either of its subtilase recognition sites was generated by means of a two-step PCR (sewing PCR) on the *GLV1* expression clone. The final PCR fragments were captured by an LR clonase reaction in the pFAST-G02 vector (Shimada *et al.*, 2010) that contains a rapid and fluorescent screenable marker, FAST, for identification of transformed Arabidopsis seeds.

### Gene expression analysis

Total RNA from 3-week-old leaves was isolated with TRIzol reagent (Invitrogen), followed by treatment with RNase-free DNase I (Qiagen) according to the manufacturer’s instructions. The cDNA was prepared with the iScript™ cDNA Synthesis Kit (Bio-Rad) from 1 μg of total RNA. For quantitative reverse transcription (RT)-PCR, 1:10 dilutions of total RNA were used (all the primers are listed in Supplementary Table S1).

### Peptide treatments

Seedlings were germinated, grown in liquid medium supplemented or not with the synthetic GLV1 peptide (GLV1p), and incubated for 6h in the light in rotating six-well plates at 21 °C, and then for 5 days in the dark under continuous rotation. The peptide was synthesized in-house as described previously (Whitford *et al.*, 2012) and dissolved in sterile 50mM sodium phosphate buffer (pH 6.0).

### Purification of the SBT6.1 enzyme from plant tissues

The myc epitope-tagged SBT6.1 protein was affinity purified from an overexpression line described previously (Srivastava *et al.*, 2009). Plantlets for protein extraction were grown in liquid ½MS medium with orbital shaking at 130rpm (Innova^TM^ 2300, New Brunswick Scientific). Fifty grams of 2-week-old Arabidopsis seedlings grown in liquid ½MS medium were ground in liquid nitrogen and suspended in ice-cold extraction buffer (25mM Tris-HCl, pH 7.6, 150mM NaCl, 0.1% Nonidet P-40, and 10% ethylene glycol) with an ultra-Turrax mixer. The supernatant was centrifuged twice at 18 000×*g*. A 100-µL volume of anti-c-myc agarose affinity gel (Sigma-Aldrich) was added to the filtered lysate and incubated for 2h at 4 °C with continuous rotation. The SBT6.1 enzyme bound to the agarose affinity gel was recovered by centrifugation at 1500×*g* for 4min at 4 °C and washed 10 times thoroughly with washing buffer (25mM Tris-HCl, pH 7.6, 150mM NaCl) through a polyprep chromatography column (Bio-Rad). The final product was resuspended in 100 µL of 25mM 2-(*N*-morpholino)-ethanesulfonic acid (MES)–sodium acetate buffer (pH 6.2). The bead-bound protein concentration was measured with the protein assay (Bio-Rad). All purified products were resolved on 10% sodium dodecyl sulfate–polyacrylamide gel electrophoresis and visualized by Coomasie brilliant blue staining or with the 9E10 monocolonal anti-myc antibody in protein immunoblots (Santa Cruz Biotechnology). For negative controls, nontransgenic plants were purified in parallel.

### Peptide assay for SBT6.1 activity *in vitro* and its inhibition by Serpin1

For protease activity assays with RALF23 and GLV1 propeptides, 19 μL of bead-bound affinity-purified myc-tagged SBT6.1 was mixed with 1 µL of a 500mM peptide solution in 25mM MES–sodium acetate buffer (pH 6.2), supplemented with 2.5mM calcium chloride, to obtain a final peptide concentration of 25 µM. Standard enzymatic reactions were incubated at 32 °C for 1h.

Serpin1 was purified from *Escherichia coli* cultures as described (Vercammen *et al.*, 2006). Of bead-bound affinity-purified myc-tagged SBT6.1, 18 µL was mixed with Serpin1 in phosphate-buffered saline/glycerol (50:50) to a final concentration of 0.5mg/mL and a total volume of 19 µL. Beads loaded with myc-tagged SBT6.1, but without Serpin1, were used as positive controls. The beads were incubated for 1h at 32 °C as described above. The SBT6.1 peptide digestion products were analysed by mass spectrometry with a matrix-assisted laser desorption ionization-time-of-flight (MALDI-TOF) spectrometer (Voyager DE STR; Applied Biosystems). The matrix contained 4–5mg α-cyano-4-hydroxycinnamic acid in 1mL acetonitrile/MilliQ water (50:50) supplemented with 10mM ammonium citrate and 1 µL trifluoroacetic acid. The crude peptide mixture was spotted on the MALDI-TOF plate and analysed.

### Analysis of SBT6.1 and Serpin1 association

*In vivo* interaction of SBT6.1 with Serpin1 was determined by tandem affinity purification (TAP) as described (Van Aken *et al.*, 2007). In brief, Arabidopsis cell suspension cultures were stably transformed by *Agrobacterium tumefaciens*-mediated cocultivation with pKNTAP-Serpin1. The TAP tag consisted of two IgG-binding domains of the *Staphylococcus aureus* protein A (ZZ) and a calmodulin-binding peptide, separated by a tobacco etch virus protease cleavage site (Rigaut *et al.*, 1999). Two-step affinity purification was done as described (Van Leene *et al.*, 2007). To increase the stringency of the data set, proteins commonly contaminating complex extracts were considered as experimental background and systematically subtracted from the lists of copurified proteins (Van Leene *et al.*, 2010).

### Histochemical and microscopic analyses

β-Glucuronidase (GUS) staining was as described previously (Beeckman and Engler, 1994). For live-cell imaging, seedlings were mounted in water with or without dye. The adaxial leaf epidermis of transfected *Nicotiana benthamiana* leaves was assayed for ﬂuorescence with a confocal microscope, LSM5 (Zeiss), equipped with ×40 and ×63 water-corrected objectives. GFP ﬂuorescence was imaged with 488-nm laser excitation. Emission ﬂuorescence was captured in the frame-scanning mode alternating GFP ﬂuorescence via a 500-/550-nm band-pass emission ﬁlter. Cell membranes of hypocotyls were counterstained with propidium iodide and imaged with a 543-nm filter and 590 to 620nm for excitation and detection, respectively.

### Transient expression in *N. benthamiana*

Wild-type (WT) *N. benthamiana* plants were grown under 14h light/10h darkness at 25 °C and 70% relative humidity. All BiFC constructs were transferred into the *A. tumefaciens* strain C58C1 harboring the virulence plasmid MP90. The obtained *Agrobacterium* strains were used to inﬁltrate the tobacco leaves, of which the transient expression was assayed. The transformed *Agrobacterium* strain harboring the constructs of interest was grown in 2mL of yeast extract broth (YEB), supplemented with appropriate antibiotics in a shaking incubator (200rpm) at 28 °C. After 1 day, 100 µL of the liquid culture was transferred to 10mL YEB supplemented with appropriate antibiotics and grown for one additional day. After incubation, the optical density at 600nm (OD_600_) of each culture was measured and the culture amount needed for OD_600_=1.5 was transferred to Eppendorf tubes and centrifuged at 6800×*g* for 5min. The bacterial pellet was resuspended in 2mL of the inﬁltration buffer (10mM MgCl_2_, 10mM MES, and 100 µM acetosyringone) and incubated for 2h as described above. For coexpression experiments, 0.33mL of each bacterial culture was mixed with the bacterial culture harboring the p19 vector to obtain 1mL of the inoculum, with each construct adjusted to a ﬁnal OD_600_=0.5. The inoculum was infiltrated through the abaxial epidermis of 3- to 4-week-old *N. benthamiana* leaves by gentle pressure with a 1-mL syringe without needle. The inﬁltrated leaf areas were delimited and labeled with an indelible pen. Plants were further grown under normal growing conditions. Four inﬁltrated leaf fragments were analysed per combination in two independent transformation events 2, 3, and 5 days after inﬁltration. Interactions were scored positive when at least 10 ﬂuorescent cells per leaf segment were observed. Infiltrated leaves were imaged with a LCS-SL CLSM confocal microscope (Leica).

### Statistical tests

Means of samples were compared with one-way or two-way analysis of variance (ANOVA) (GraphPad Prism; V6.00, GraphPad Software). Data were pooled from two independent biological replicates, unless specified otherwise.

## Results

### Specific subtilases are necessary for GLV1 peptide signaling

**Fig. 1.**
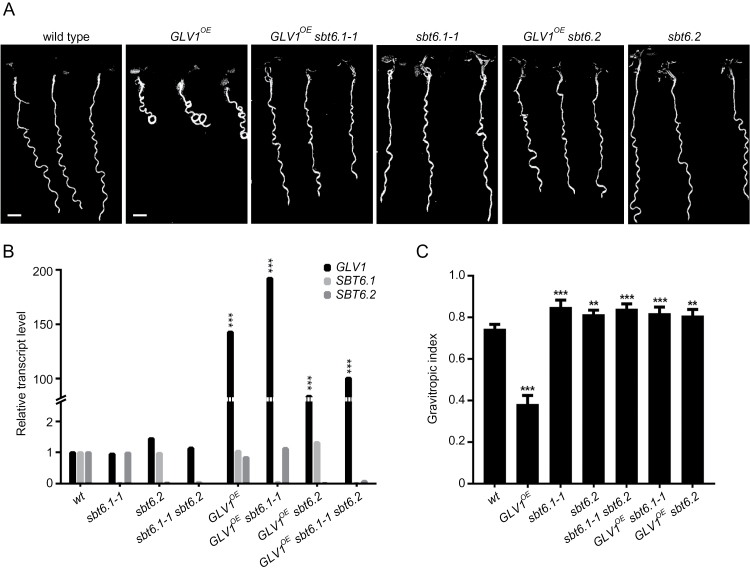
Suppression of the *GLV1* ^*OE*^ curly root phenotype in subtilase mutants. (A) Seedlings grown on inclined agar plates for 7 days after germination (dag). Scale bars: 2mm. (B) Relative transcript levels compared with WT as measured by quantitative RT-PCR analysis (mean transcript level ± confidence interval [CI]; one-way ANOVA). (C) Gravitropic index (mean GI index ± CI compared with WT; one-way ANOVA, *n*=18–39). Error bars represent the 95% confidence interval. Asterisks mark significant differences: *P<0.05; **P<0.005; ***P<0.001.

Gain-of-function *GLV1* seedlings have an agravitropic curly root when grown on an inclined agar surface (Whitford *et al.*, 2012). Therefore, if subtilases were responsible for processing of the GLV1 propeptide into its bioactive form, the agravitropic gain-of-function phenotype should be suppressed by a mutation in the corresponding *SBT* gene. Based on this assumption, the *GLV1* gene under the control of the *35S* promoter of the cauliflower mosaic virus was transformed into 74 Arabidopsis T-DNA insertion lines, in which 55 of the 56 identified subtilase genes had been mutated (Supplementary Table S2) (Rautengarten *et al.*, 2005). The resulting T1 plants were grown on slanted plates and their root phenotypes were scored. Three mutant alleles representing two genes, namely *SBT6.1* (site-1 peptidase or AtS1P; MEROPS ID S08.063; At5g19660) and *SBT6.2* (tripeptidyl-peptidase II; MEROPS ID S08.090; At4g20850), suppressed the agravitropic root phenotype caused by the *GLV1* gain-of-function (Fig. 1A). These subtilase mutants had been identified as SALK_111474 (hereafter designated *sbt6.1-1*), SALK_020530 (*sbt6.1–2*), and SALK_085776 (*sbt6.2*), each carrying a T-DNA insert into an exon (Rautengarten *et al.*, 2005) (Supplementary Table S2).

Several independent *GLV1* ^*OE*^ homozygous lines were obtained for each of the three *sbt* mutant genotypes and those with a high level of *GLV1* transcripts were selected for further study. In all cases, quantitative RT-PCR analysis confirmed that the suppression was linked to the lack of expression of the *SBT6.1* or *SBT6.2* subtilase gene and that in the selected transformed lines the *GLV1* gene was overexpressed at levels that cause agravitropic root growth in WT plants (Fig. 1B and Supplementary Table S3).

Root growth phenotypes were quantified by measurement of the gravitropic index (GI), which is the ratio between the primary root length and the linear distance separating the collet from the root tip (Fig. 1C) (Grabov *et al.*, 2005). Comparative analysis confirmed that the strong gravitropic defect of *GLV1* ^*OE*^ roots is suppressed in the *sbt6.1-1* and *sbt6.2* mutants, but also revealed that the *SBT6* loss-of-function alone resulted in a phenotype opposite to that of the *GLV1* gain-of-function: the single *sbt6.1-1* and *sbt6.2* lines and the double loss-of-function *sbt6.1-1 sbt6.2* line had a GI that was higher than that of the WT. These results suggest that SBT6.1 and SBT6.2 are necessary for the processing of GLV1 and, possibly, of other GLV precursors, because mature bioactive GLV peptides are involved in root gravitropic responses (Whitford *et al.*, 2012).

### GLV and SBT6 functions interact to control hypocotyl elongation

**Fig. 2.**
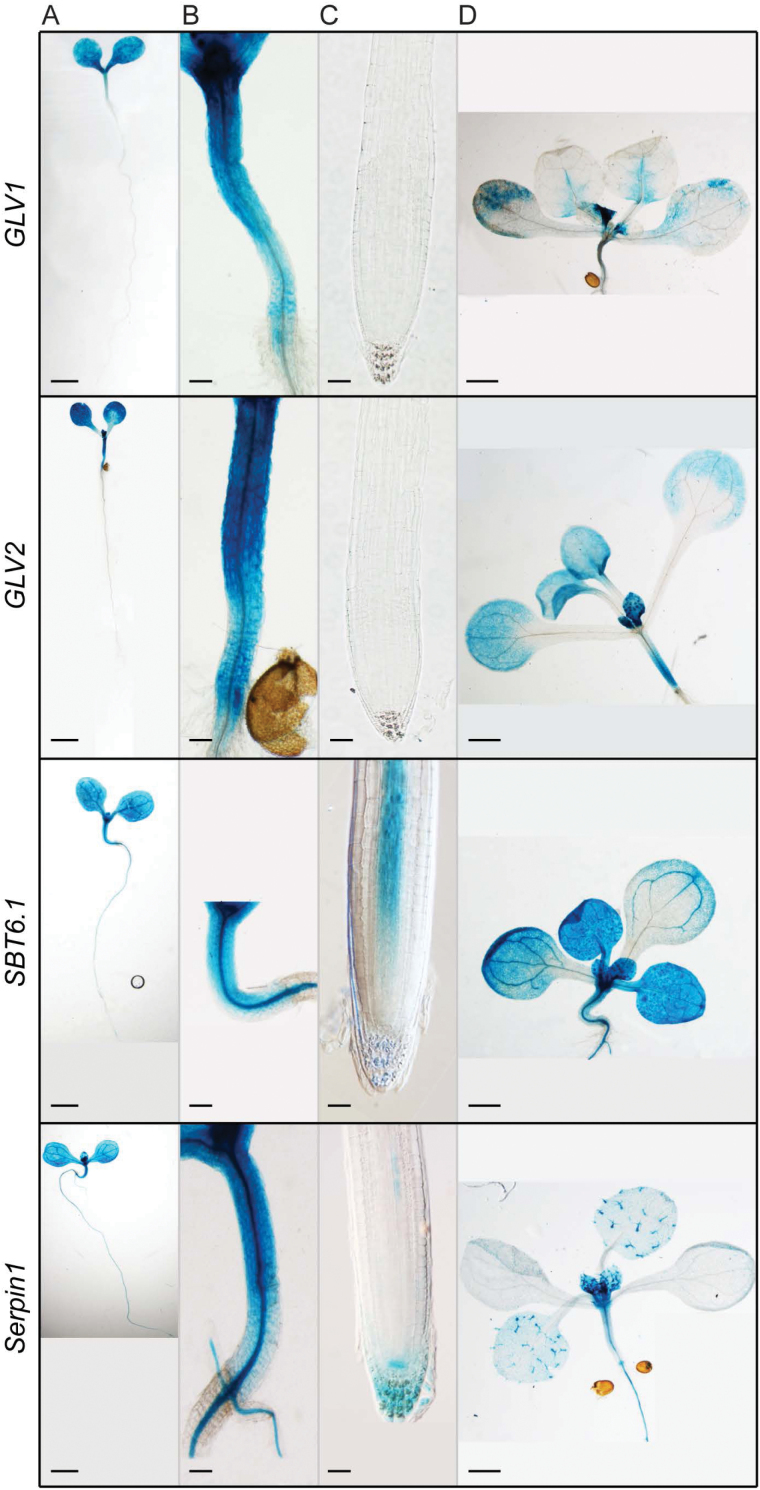
Transcriptional activity of *GLV1*, *GLV2*, *SBT6.1*, and *Serpin1*. (A–C) Young seedling, hypocotyl, and root tip (5 dag). (D) Cotyledons and first leaves (10 dag). Plants were transformed with the corresponding *promoter:GUS* transgenes. Scale bars: 1mm (A and D), 100 µm (B), and 50 µm (C).

**Fig. 3.**
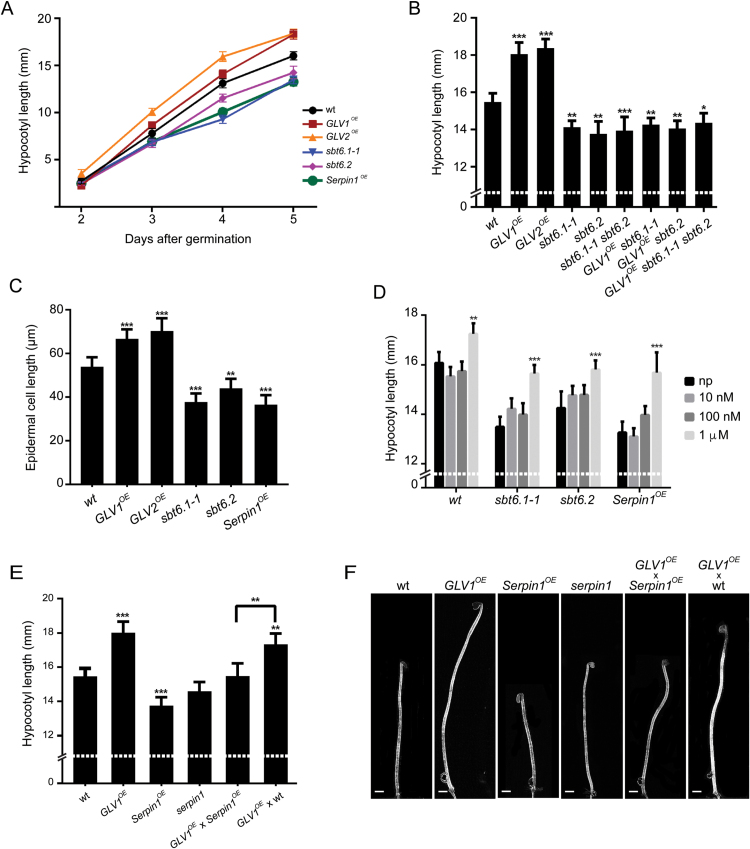
Hypocotyl elongation phenotypes. (A) Kinetics of etiolated hypocotyl growth (mean hypocotyl length in millimeters ± CI; two-way ANOVA; *n*=32–80). (B) Hypocotyl length 5 dag (mean hypocotyl length in millimeters ± CI compared with WT; one-way ANOVA; *n*=32–65). (C) Hypocotyl epidermal cell length (mean of the three most elongated cells from each seedling in micrometers, one-way ANOVA; *n*=15). (D) Increased hypocotyl length (in millimeters) upon GLV1p treatment. Peptide treatments at different concentrations were compared with mock-treated (without peptide; np) plants of the same genotype 5 dag (two-way ANOVA; *n*=52–100). Error bars represent the 95% confidence interval. Asterisks mark significant differences: *P<0.05, **P<0.005, ***P<0.001. (E) Hypocotyl length at 5 dag (mean hypocotyl length in millimeters ± CI compared with WT; one-way ANOVA; *n*=20–61). Hemizygous F1 plants were measured to assess the interaction between the *GLV1* and *AtSerpin1* gain-of-function in comparison with F1 plants resulting from a cross between *GLV1* ^*OE*^ and WT plants. (F) Representative hypocotyl length. Error bars represent the 95% confidence interval. Asterisks mark significant differences: *P<0.05, **P<0.005, ***P<0.001. Scale bars: 1mm.

Whereas the screen was based on a root phenotype due to overexpression, the native *GLV1* gene together with its close homolog *GLV2* are not transcribed in the root, but in the aerial part of Arabidopsis plants, including the growing hypocotyl (Whitford *et al.*, 2012; Fernandez *et al.*, 2013*b*) (Fig. 2), as is also *SBT6.1* (Fig. 2) (Liu *et al.*, 2007). Based on their common expression domain, these three genes were hypothesized to be involved in hypocotyl development. Compared with the WT, the elongation of *GLV1* ^*OE*^ and *GLV2* ^*OE*^ hypocotyls grown in the dark was faster, whereas that of *sbt6.1-1*, *sbt6.2*, and *sbt6.1-1 sbt6.2* was slower (Fig. 3A, B). Furthermore, the increase in hypocotyl size induced by *GLV1* ^*OE*^ or *GLV2* ^*OE*^ was suppressed in the *sbt6* loss-of-function mutants (Fig. 3B). The difference in hypocotyl growth between the genotypes was not the indirect consequence of early developmental delays, because all lines germinated simultaneously and their hypocotyl length was undistinguishable at the beginning of the experiment (Fig. 3A). Thus, these observations indicate that the subtilases positively control hypocotyl elongation, possibly through GLV peptide processing.

Single loss-of-function of the artificial microRNA interference knockdown *amiRglv1* and T-DNA knockout *glv2-1* lines and the double *amiRglv1 glv2-1* line had no significant defect in hypocotyl growth (data not shown), probably because of the partially redundant action of the *GLV* genes. For example, the *GLV10* transcript was detected in the growing hypocotyl (Fernandez *et al.*, 2013*b*). Alternatively, additional non-GLV signaling peptide precursors that positively regulate hypocotyl elongation may also need to be processed by the subtilases to become active.

The length of hypocotyl epidermal cells, where *GLV1* and *GLV2* are primarily transcribed, was measured in *GLV* ^*OE*^ and *sbt6* mutant lines (Supplementary Fig. S1) (Whitford *et al.*, 2012). The cells were longer in plants overproducing the GLV1 or GLV2 peptide and shorter in the *sbt6.1-1* and *sbt6.2* lines than those of the WT (Fig. 3C and Supplementary Fig. S1). The results imply that GLV signaling positively regulates hypocotyl growth by promoting cell elongation and that this control may depend on the SBT6 subtilase activity.

### The GLV1 peptide promotes hypocotyl elongation and rescues the sbt6 phenotype

The bioactive peptide is encoded in a conserved C-terminal motif of the GLV1 precursor (Whitford *et al.*, 2012). To confirm that the GLV1-induced hypocotyl growth can be attributed to that domain, Arabidopsis seedlings were treated with a synthetic GLV1p peptide similar to the mature native signal, DY(SO_3_H)PQPHRKPPIHNE, with Y(SO_3_H) indicating a sulfated tyrosine. Hypocotyls of plants incubated for 5 days in liquid ½MS medium supplemented with 1 µM GLV1p were longer than those in control plants (Fig. 3D).

If SBT6 subtilases were involved in the GLV1 precursor processing, then the *sbt* loss-of-function mutants should still respond to the addition of the GLV1 mature peptide. To test this assumption, the GLV1p impact on *sbt6.1* and *sbt6.2* plants was measured: the hypocotyl length of the GLV1p-treated mutants was longer than that of the untreated counterparts, reaching a size undistinguishable from that of untreated WT control plants (Fig. 3D). This peptide effect together with the failure of *GLV1* ^*OE*^ plants to produce increased hypocotyls in the *sbt6.1* and *sbt6.2* backgrounds indicates that the subtilases act upstream of the GLV signal perception.

### GLV precursors are proteolytically cleaved by SBT6.1

**Fig. 4.**
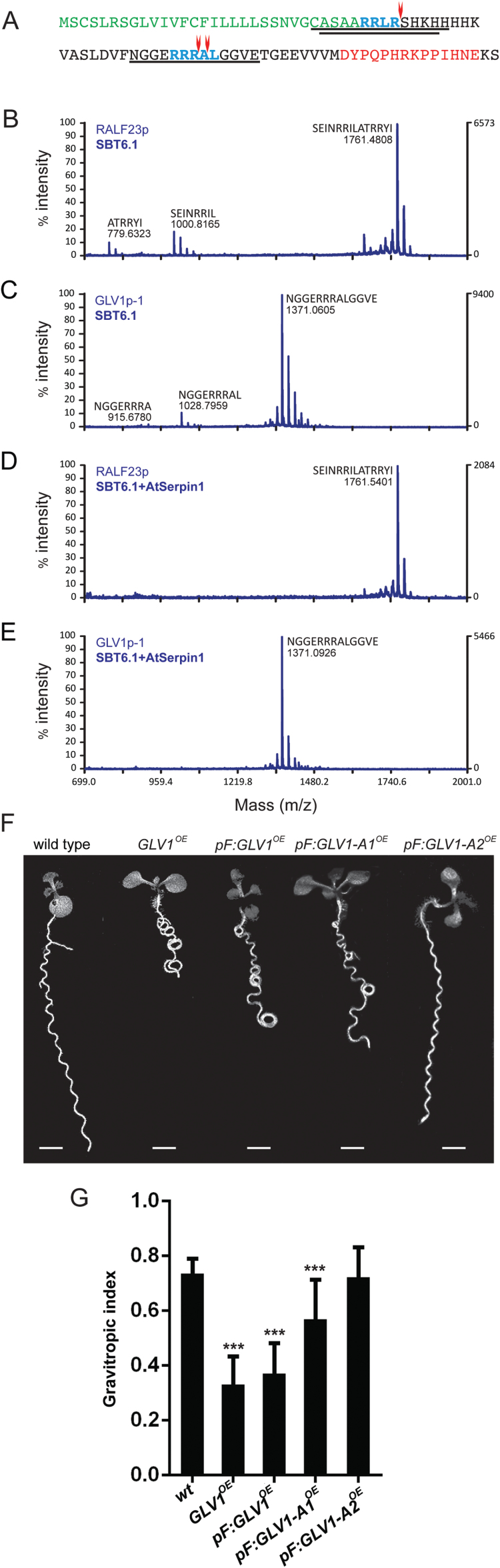
*In vitro* proteolytic activity of SBT6.1 and suppression of the *GLV1* ^*OE*^ curly root phenotype by mutation at the subtilase recognition motifs. (A) GLV1 propeptide sequence. Signal peptide, green; subtilase canonical cleavage site, blue; GLV motif, red; observed cleavage sites, red arrow; tested synthetic peptides, underlined. (B–E) Synthetic peptide and fragments after 1h of incubation with SBT6.1 for RALF23 (B and D) and GLV1 (C and E) with (D and E) or without (B and C) AtSerpin1. (F) Seedlings grown on inclined agar plates for 7 dag. Scale bars: 2mm. *pF:GLV1-A1* and *pF:GLV1-A2*, lines overexpressing the *GLV1* gene carrying mutations at the first and second subtilase recognition site, respectively. (G) Gravitropic index (mean GI index ± CI compared with WT; one-way ANOVA, *n*=40–67). Error bars represent the 95% confidence interval. Asterisks mark significant differences: ***P< 0.001.

To investigate whether the SBT6 proteolytic activity might be involved directly in the processing of the GLV1 precursor protein, the myc epitope-tagged SBT6.1 protein was overproduced in Arabidopsis (Srivastava *et al.*, 2009) and the subtilase was affinity purified from extracts of whole plants germinated and grown in liquid medium. Two canonical subtilase recognition sequences were identified within the variable region of the GLV1 precursor (Fig. 4A). The SBT6.1 enzyme bound to anti-myc beads was incubated with synthetic propeptide fragments that corresponded to these regions (Fig. 4A and Supplementary Table S4). As a control, the subtilase proteolytic activity was confirmed with a synthetic RALF23 propeptide fragment that is a known SBT6.1 substrate (Fig. 4B and Supplementary Table S4) (Srivastava *et al.*, 2009). The enzymatic digestion products were analysed by MALDI-TOF mass spectrometry. The tested GLV1 propeptide-derived products revealed two SBT6.1 *in vitro* cleavage sites after the sequences RRLR (Site1) and RRRAL (Site2), and a minor one ending with RRRA (Fig. 4A, C, Supplementary Fig. S3 and Supplementary Table S4).

As specified previously, gain-of-function *GLV1* seedlings have an agravitropic curly root when grown on an inclined agar surface (Whitford *et al.*, 2012). It is believed that subtilases distinguish specific recognition motifs in their target precursors and cleave them at or near these motifs. Therefore, if subtilases were responsible for the processing the GLV1 propeptide into its bioactive form, the agravitropic gain-of-function phenotypes should be suppressed by abolishing the subtilase recognition motifs. To investigate *in vivo* processing of the GLV1 precursor protein by SBT6, *GLV1* ^*OE*^ mutant lines were generated that carried mutations in one of the two subtilase recognition motifs. In these lines, stretches of four amino acids in either of the subtilase recognition motifs (*RRLR* and R*RRAL*; Fig. 4A) were replaced by alanine residues in the mutant GLV1-overproduced propeptide, designated GLV1-A1 and GLV1-A2 for the mutated Site1 and Site2, respectively. Several independent *GLV1* ^*OE*^ T1 lines were obtained for each of the mutant proteins and those with high levels of mutant *GLV1* transcripts were selected for further study.

The resulting T1 plants were grown on slanted plates and their root phenotypes were scored (Fig. 4F, G). Root growth phenotypes were quantified by GI index. Comparative analysis confirmed that alanine substitutions in the subtilase recognition motifs reduced the GLV1 agravitropic gain-of-function phenotypes. Mutations at the Site2 subtilase recognition motif strongly suppressed the *GLV1* gain-of-function phenotypes and *GLV1-A2* ^*OE*^ showed no significant differences with the WT, whereas mutation at the Site1 subtilase recognition motif only partially reduced the *GLV1* agravitropic gain-of-function phenotype, but was still significantly different from the WT.

In summary, SBT6.1 cleaved the GLV1 precursor protein *in vitro* and SBT6.1-processed sites were required for GLV1 activity *in vivo*. Furthermore, null mutations in *SBT6.1*, and its closest homolog, *SBT6.2*, suppressed the *GLV1* gain-of-function phenotypes. Together, *in vivo* and *in vitro* results indicate that the SBT6 processing is needed for maturation and activation of the GLV1 propeptide.

### SBT6.1 associates with the Serpin1 protease inhibitor

The activity of proteases involved in the control of developmental processes has to be tightly regulated (Turk, 2006). In a separate TAP pull-down experiment, aimed at identifying interactors of the Serpin1 protease inhibitor (MEROPS ID I04.087; also referred to as AtSerpin1), we identified that SBT6.1 forms *in vivo* protein complexes with Serpin1 (Supplementary Table S5), suggesting that SBT6.1 activity may be regulated by a protease inhibitor.

**Fig. 5.**
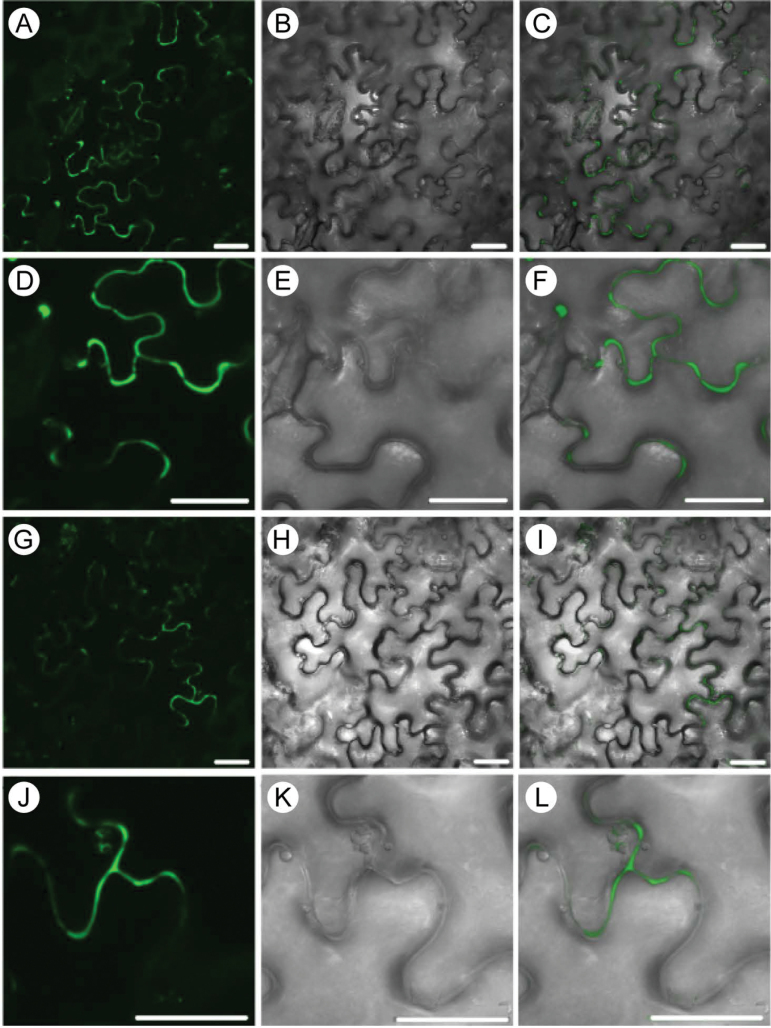
BiFC interaction between Serpin1 and SBT6.1. (A–F) Interaction between SBT6.1-nGFP and Serpin1-cGFP. (G–L) Interaction between SBT6.1-cGFP and Serpin1-nGFP. The subcellular localization was determined in the leaf epidermis of *N. benthamiana* (A–I). GFP fluorescence (A, D, G, and J), Nomarski differential interference contrast (DIC) (B, E, H, and K), and GFP/DIC overlapping images (C, F, I, and L). All images resulted from stacked confocal sections. Scale bars: 25 µm.

The close association between SBT6.1 and Serpin1 was confirmed by BiFC analysis (Boruc *et al.*, 2010). Both proteins were transiently coproduced as translational fusions with the truncated EGFP halves in epidermal cells of *A. tumefaciens*-transfected *N. benthamiana*. SBT6.1 was fused to the N terminus of EGFP (nGFP) and combined with the Serpin1 protein that had been fused to the C terminus of EGFP (cGFP), and vice versa. In all cases, the EGFP fragments were fused at the C end of the tested interactors. In both configurations, AtSBT6.1 and Serpin1 interactions resulted in strong apoplastic signals (Fig. 5). As negative controls, single constructs (either Serpin1 or SBT6.1 fused to nGFP or cGFP) were tested, but without any detectable signal.

To investigate whether the association of SBT6.1 with a protease inhibitor negatively regulated the subtilase proteolytic activity, the cleavage of the GLV1 precursor sequences by the SBT6.1 enzyme was tested in the presence of Serpin1. As expected, no digested peptide could be detected by MALDI-TOF when Serpin1 was added to the purified SBT6.1 protein (Fig. 4D, E). These results demonstrated that Serpin1 inhibits the SBT6.1 activity *in vitro*.

### Serpin1 overexpression suppresses GLV-dependent hypocotyl elongation

The biochemical analysis pointed toward a potential role of Serpin1 in GLV-dependent regulation of hypocotyl elongation through the control of the SBT6.1 proteolytic activity. In agreement with this model, the *Serpin1* promoter was active in hypocotyls as well as in other plant parts (Fig. 2; Supplementary Fig. S2).

The hypocotyls of *Serpin1*-overexpressing (*Serpin1* ^*OE*^) plants were shorter and their epidermal cells smaller than those of WT plants (Fig. 3C–E). These phenotypes were reminiscent of those observed in *sbt6.1-1* and *sbt6.2* loss-of-function mutants (compare Fig. 3E, F with Fig. 3B, D). Furthermore, the long-hypocotyl phenotype associated with the *GLV1* gain-of-function was suppressed in *Serpin1* ^*OE*^ seedlings (Fig. 3E, F). Finally, *Serpin1* ^*OE*^ seedlings treated with the bioactive synthetic GLV1p had longer hypocotyls than the untreated seedlings, with a response similar to that of *sbt6.1-1* and *sbt6.2* loss-of-function mutants, thereby confirming that the SBT6 and Serpin1 activities are involved in the production of the GLV signal (Fig. 3D).

## Discussion

### The catalytic processing of subtilases is required for GLV peptide production

The initial suppressor screen based on *GLV1* ^*OE*^ root phenotypes and the subsequent analysis of related hypocotyl growth phenotypes revealed that the genes coding for SBT6.1 and SBT6.2 are necessary for the maturation and activation of the GLV1 peptide. These two proteins are most closely related to each other in the subtilase phylogenetic tree and may, therefore, have similar activities (Rautengarten *et al.*, 2005). However, recessive null mutations in either gene resulted in undistinguishable phenotypes and the *sbt6.1-1 sbt2* double mutant did not exhibit an additive phenotype, suggesting that the two subtilases act instead at successive stages during GLV1 maturation.

*In vitro* protease assays showed that the plant-purified SBT6.1 enzyme cleaves GLV1 precursor peptides at sites reminiscent of the canonical recognition sequences for subtilases, RXXL and RXLX (Schaller *et al.*, 2012). As 10 out of 11 GLV precursors carry at least one of these sites (Supplementary Table S6), SBT6.1 may cleave multiple members of the GLV family. The majority of the GLV peptides occur in root tissues, some of which are involved in root gravitropic responses (Whitford *et al.*, 2012; Fernandez *et al.*, 2013*b*). That the s*bt6.1* and *sbt6.2* loss-of-function mutants have a higher gravitropic index than the WT hints at the involvement of *SBT6.1* and *SBT6.2* in the maturation of GLV precursors produced in the primary Arabidopsis root, other than GLV1, which is not produced in the root.

*In vivo* alanine replacement experiments showed that the recognition motifs in the amino acid sequence of the GLV1 precursor are important for subtilase-related processing events. These data show that appropriate activity of the *SBT6.1* gene and recognition of the target sequence in signaling peptide precursors highly depends on the correct substrate recognition sequence.

Finally, the SBT6.1 cleavage sites identified in the GLV1 precursor sequence are not sufficient to produce the mature peptide detected in plant tissues (Whitford *et al.*, 2012). The additional processing steps are probably catalysed by other proteases, such as SBT6.2, the homolog of the mammalian tripeptidyl peptidase TPPII of which the proteolytic activity is quenched by TPPII-specific inhibitors (Book *et al.*, 2005). Carboxypeptidases may also be involved, such as SUPPRESSOR OF LLP1 1, for example, which cleaves C-terminal lysine and arginine residues off the end of CLE peptides and is required for CLE19 signaling (Casamitjana-Martínez *et al.*, 2003; Tamaki *et al.*, 2013).

### Protease inhibitors as peptide signaling modulators

Among the 68 peptidase inhibitor families, the serpins are one of the two largest groups that can be found in all kingdoms. Serpins are suicide inhibitors that form irreversible covalent complexes with their targets (Huntington *et al.*, 2000). Their function in plants remains poorly understood (Fluhr *et al.*, 2012). Thus far, the Arabidopsis *Serpin1* has been shown to be involved in the inhibition of the Arabidopsis metacaspase 9 and the vacuolar protease RESPONSIVE-TO-DESICCATION21 (Vercammen *et al.*, 2006; Lampl *et al.*, 2010, 2013).

**Fig. 6.**
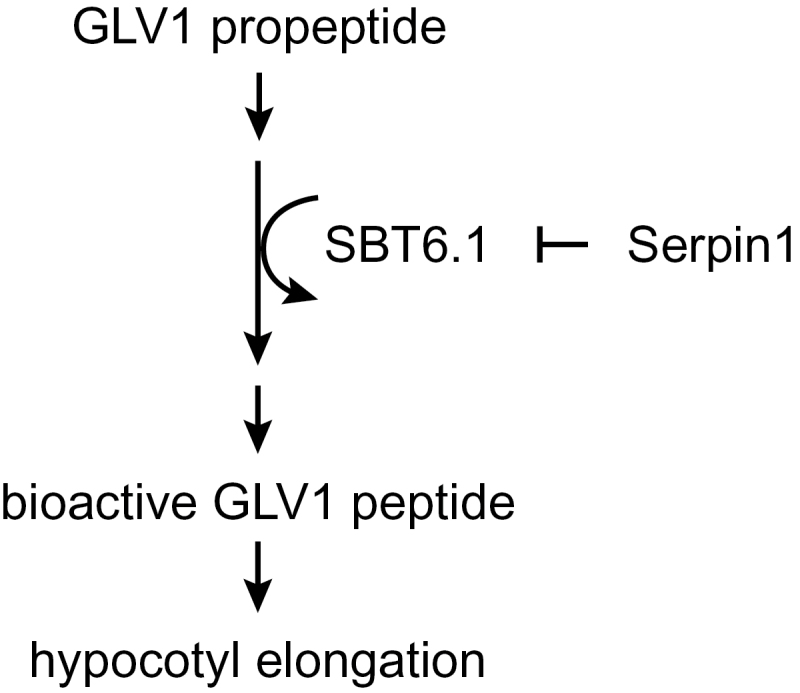
Model for *GLV*-dependent hypocotyl elongation.

Our data indicate that SBT6.1 is also under the control of the Serpin1 protease inhibitor: protein complex and BiFC analyses have shown that both proteins interact and that the proteolytic activity of the subtilase is inhibited by Serpin1 *in vitro*. Furthermore, *Serpin1* ^*OE*^ phenocopies *sbt6.1* and *sbt6.2* null mutants and suppresses *GLV1* gain-of-function. These observations can be summarized in a model in which hypocotyl cell elongation is positively regulated by the GLV1 peptide, whereas its production is catalysed by SBT6.1, which is itself inhibited by Serpin1 (Fig. 6).

Serpins carry a reactive center loop, including a protease target sequence as bait. Upon cleavage of this target sequence, the reactive center loop undergoes an irreversible conformational change that locks and inactivates the protease (Lampl *et al.*, 2010). Serpin1 contains the canonical cleavage site RGLL, which makes it a potential target for SBT6.1, in agreement with the protein interaction and biochemical analyses of the SBT6.1 proteolytic activity.

The SBT6.1 protein has been located in the Golgi apparatus (Liu *et al.*, 2007) and Serpin1 has been detected in the cytosol, Golgi bodies, endoplasmic reticulum, and apoplast (Vercammen *et al.*, 2006; Lampl *et al.*, 2013). Therefore, SBT6.1 and Serpin1 can interact to regulate GLV-dependent hypocotyl growth. Nevertheless, because SBT6.1 is not the sole target of Serpin1, it cannot be excluded that the inhibiting effect on the hypocotyl growth might also be partly relayed through inactivation of other proteases.

The 11 Arabidopsis *GLV* genes share sequence similarity, but they are expressed specifically in different tissues (Whitford *et al.*, 2012; Fernandez *et al.*, 2013*b*), in contrast to the relatively broad expression patterns of the *SBT6.1* and *Serpin1* genes in Arabidopsis. Therefore, they may be involved in the coregulation of GLV functions in various tissues, including, but not exclusively, in the hypocotyl. For example, 7 out of 11 *GLV* genes are transcribed in the root tip (Fernandez *et al.*, 2013*b*), where *SBT6.1* and *Serpin1* are expressed as well (Supplementary Fig. S1). Whereas *Serpin1* is transcribed in all tested organs (Ahn *et al.*, 2009), its expression is seemingly not uniform across all cell types (Supplementary Fig. S2). Hence, by limiting the SBT6.1 activity, Serpin1 might contribute to the fine spatial regulation of the GLV peptide biosynthesis.

### GLV peptides control hypocotyl elongation together with other secreted peptides

The experimental results demonstrate that GLV signals promote cell elongation in the growing hypocotyl: (i) overexpression of the *GLV1* and *GLV2* genes, normally transcribed in the outer cell layers of the hypocotyl (Whitford *et al.*, 2012), results in increased epidermal cells size; (ii) application of the bioactive synthetic GLV1p enhances hypocotyl length; and (iii) null mutations in genes coding for subtilases necessary for the proteolytic processing of the GLV1 precursor cause a short-hypocotyl phenotype. These observations confirm that *GLV1* and *GLV2* play a positive role in regulation of the cell expansion, as already suggested by their requirement for the gravitropic responses of reoriented hypocotyls (Matsuzaki *et al.*, 2010).

Albeit highly significant, the differences observed in hypocotyl lengths are limited to 10–20% gain or loss when compared with those of WT, indicating that other signals take part in the control of the hypocotyl growth as well. In fact, other secreted peptides have been shown to promote hypocotyl cell expansion, including PSK-α and PLANT PEPTIDE CONTAINING SULFATED TYROSINE 1 (PSY1) (Amano *et al.*, 2007; Stührwohldt *et al.*, 2011; Hartmann *et al.*, 2013). Conspicuously, mature GLV, PSK, and PSY peptides all carry a sulfated tyrosine residue that is important for bioactivity and results from the activity of the tyrosylprotein sulfotransferase (Matsubayashi, 2014) and they may share other processing enzymes, including subtilases. Yet, a possible crosstalk between the peptide signaling pathways driving cell expansion remains to be elucidated, as well as their connection with hormonal growth control.

## Supplementary data

Supplementary data are available at *JXB* online.

Figure S1. Hypocotyl elongation phenotypes.

Figure S2. Transcriptional activity of pSerpin1:GUS.

Figure S3. MALDI-TOF spectra for synthetic peptides.

Table S1. Primers used to confirm overexpression and knockout lines.

Table S2. Subtilase (*sbt*) mutant genotypes of *Arabidopsis*.

Table S3. *GLV1* transcript fold induction in transformed *sbt6* T-DNA mutant lines.

Table S4. Propeptides and observed proteolytic products according to *m/z* for singly charged ions.

Table S5. Proteins identified after TAP purification with NTAP-Serpin1 expressed in *Arabidopsis* cell suspension cultures.

Table S6. Typical subtilase target sequences in GLV peptides.

Supplementary Data

## Abbreviations:

½MS: half-strength Murashige and Skoog
ANOVA: analysis of variance
BiFC: bimolecular fluorescence complementation
dag: days after germination
EGFP: enhanced green fluorescent protein
GI: gravitropic index
GLV: GOLVEN
GUS: β-glucuronidase
MALDI-TOF: matrix-assisted laser desorption ionization-time-of-flight
OD_600_: optical density at 600 nm
PCR: polymerase chain reaction
PSK: phytosulfokine
RALF: rapid alkalinization factor
SBT: subtilase
TAP: tandem affinity purification
WT: wild-type
YEB: yeast extract broth.
