# Supplementary material for: The SBT6.1 subtilase processes the GOLVEN1 peptide controlling cell elongation
Source: J Exp Bot. 2016 Jun 16;67(16):4877–87. doi: 10.1093/jxb/erw241 (PMC4983112; doi:10.1093/jxb/erw241)
Supplement: Supplementary Data [file supp_67_16_4877__index.html]

The SBT6.1 subtilase processes the GOLVEN1 peptide controlling cell elongation — The SBT6.1 subtilase processes the GOLVEN1 peptide controlling cell elongation — Supplementary Data 

# The SBT6.1 subtilase processes the GOLVEN1 peptide controlling cell elongation

## Supplementary Data

Data files

- supplementary\_figures\_S1\_S3\_Tables\_S1\_S6.pdf - Supplementary Data
