# Supplementary material for: The SBT6.1 subtilase processes the GOLVEN1 peptide controlling cell elongation
Source: J Exp Bot. 2016 Jun 16;67(16):4877–87. doi: 10.1093/jxb/erw241 (PMC4983112; doi:10.1093/jxb/erw241)
Supplement: Supplementary Data [file supp_erw241_supplementary_figures_S1_S3_Tables_S1_S6.pdf]

SUPPLEMENTARY DATA

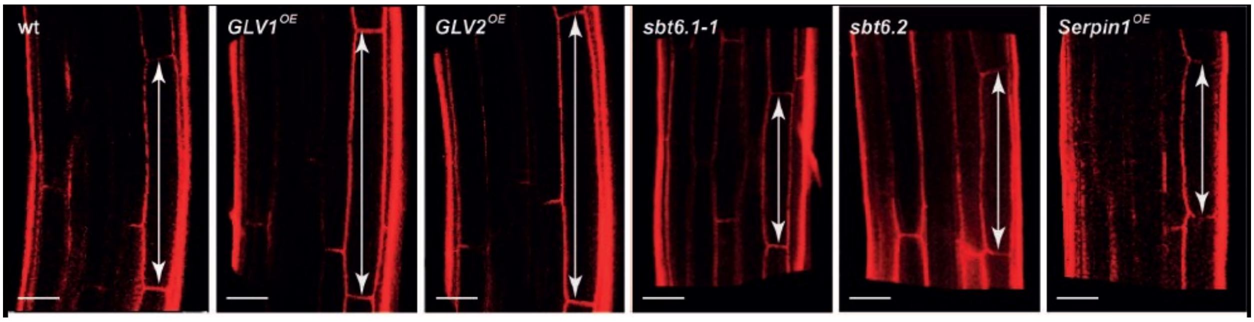

Fig. S1. Hypocotyl elongation phenotypes.

Comparison of hypocotyl epidermal cells. For all genotypes, the imaged cells were the most elongated in the light-grown seedlings (5 dag). Scale bars=10 μm.

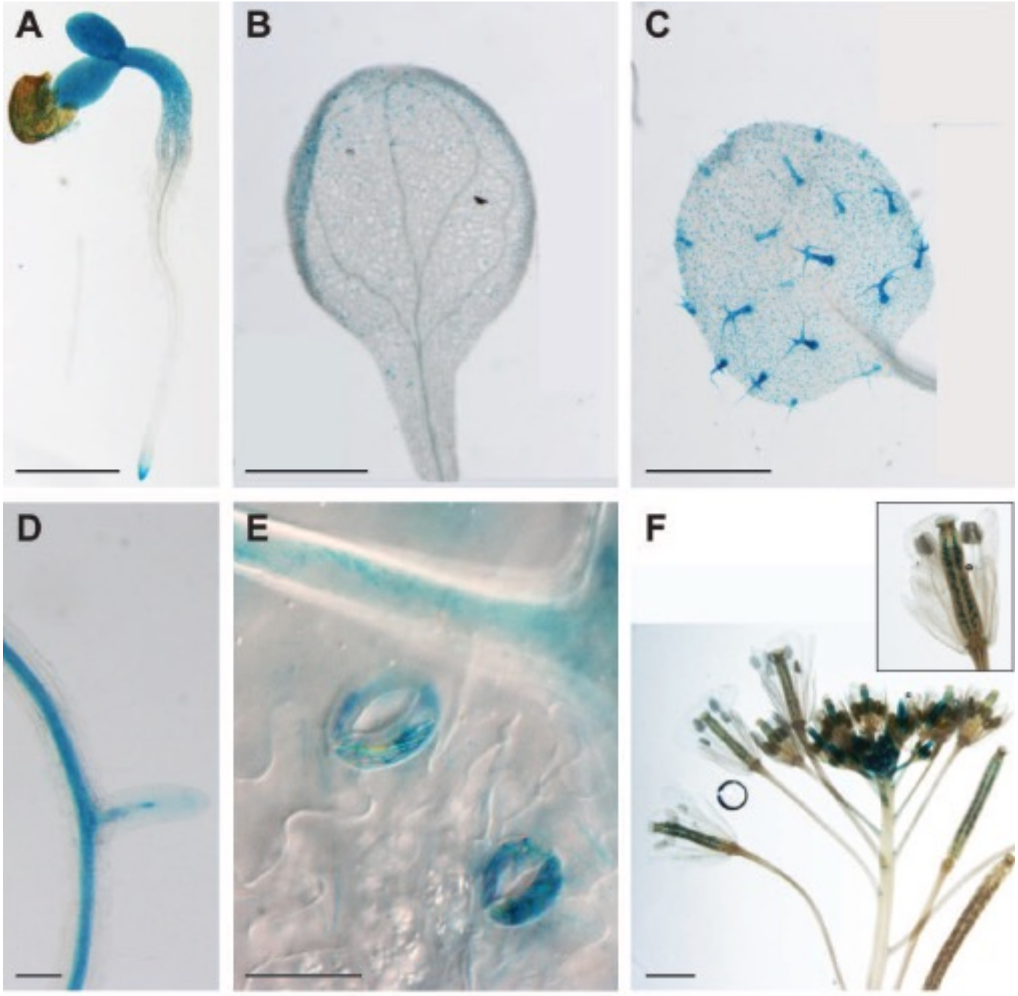

Fig. S2. Transcriptional activity of pSerpin1:GUS.

(A) Young seedling (3 dag). (B-F) Cotyledon and first leaves, trichomes, stomata, lateral root, and inflorescence (10 dag), respectively. Scale bars=1 mm (A-D and F), 25 μm (E).

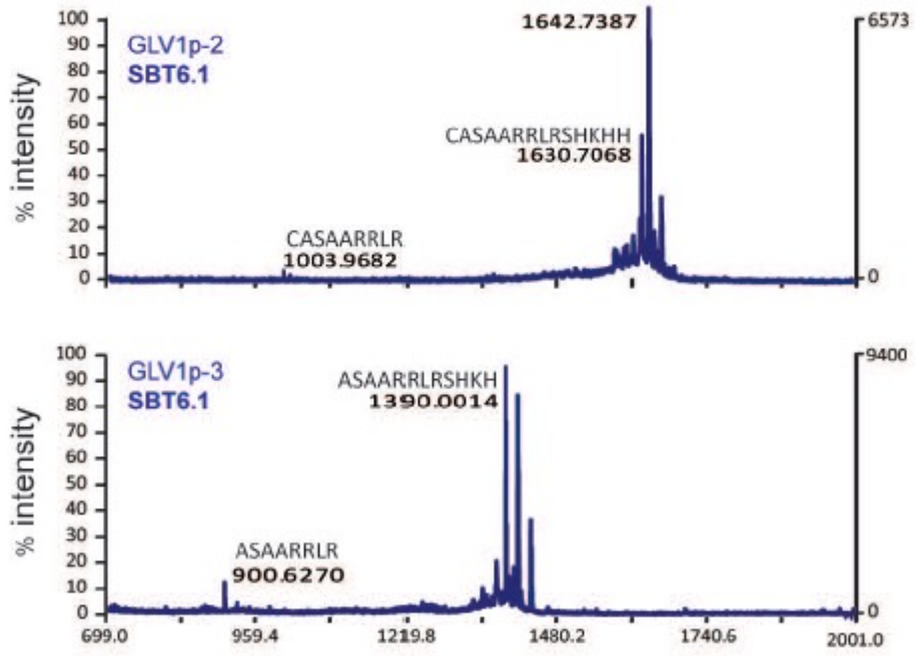

Fig. S3. MALDI-TOF spectra for synthetic peptides.

Synthetic GLV1-2 and GLV1-3 peptides (Table S3) and fragments after 1 h of incubation with SBT6.1.

Table S1. Primers used to amplify and/or confirm overexpression and knockout lines

Primer name	Sequence 5′–3′
Sbt6.1 LP	AGCGTACGAATTGGACAAATG
Sbt6.1 RP	AGGACCTGAAAGCTTAGCAGC
Sbt6.2 LP	ATTGAGGAACTGAGCAAATGG
Sbt6.1 RP	AGAAGTCTGCTAGTTTCCCGC
LBb1.3	ATTTTGCCGATTTCGGAAC
GLV1 F	ATGTATGTTGAATGTAAAAT
GLV1 R	AGACTTCTCGTTGTGGATCG
35S F	CCACTATCCTTCGCAAGACCCTTCC
qPCR-Sbt6.1 F	CCACCCCCGGGCAAGCATTT
qPCR-Sbt6.1 R	TGCAGGGTGCCATGTTGGTGG
qPCR-Sbt6.2 F	TCAAGCCGGGGGCCAACATC
qPCR-Sbt6.2 R	CGCAATTGCCCCACAGGCAGA
pSerpin1 F	GGGGACAAGTTTGTACAAAAAAGCAGGCTTAGTTAGTGTCATAATATCAAATG
pSerpin1 R	GGGGACCACTTTGTACAAGAAAGCTGGGTTTTCGCCGGAGGTTGTGGTG
pRRLR Fwd	gccgccgccgccTCACATAAGCATCATCATCAC*
pRRLR Rev	ggcggcggcggcAGCGGCAGAGGCACATCC*
pRRRAL Fwd	gccgccgccgccgccGGTGGAGTCGAGACAGGGGAAG*
pRRRAL Rev	ggcggcggcggcggcTTCTCCACCATTGAAGACGTC*

* Lowercase letters defines subtilases recognition motifs and uppercase letters represent flanking sequences.

Table S2. Subtilase (*sbt*) mutant genotypes of *Arabidopsis*

Gene name	AGI code	Mutant ID	FST position	Homozygous	Heterozygous	Suppression of agravitropic root phenotype
AtSBT1.1	At1g01900	SALK_033704	Exon	+		No
		SALK_017912	Exon	+		No
AtSBT1.2	At1g04110	SALK_035559	300-UTR-5'	+		No
AtSBT1.3	At5g51750	SALK_011867	Exon		+	No
AtSBT1.4	At3g14067	SALK_054778	Exon	+		No
		SALK_063823	Exon	+		No
AtSBT1.5	At3g14240	SALK_032651	Exon	+		No
AtSBT1.6	At4g34980	GK_270F06	Exon	+		No
AtSBT1.7	At5g67360	GK_140B02	Exon	+		No
AtSBT1.8	At2g05920	GK_168E04	Exon	+		No
		SALK_020799	Exon	+		No
AtSBT1.9	At5g67090	SALK_009925	Exon		+	No
		SALK_009917	Exon	+		No
		GK_100G11	300-UTR-3'	+		No
AtSBT2.1	At1g30600	GK_202H08	Exon	+		No
		SALK_091134	Exon	+		No
AtSBT2.2	At4g20430	SALK_150020	Exon	+		No
		SALK_013152	Exon	+		No
AtSBT2.3	At5g44530	GK_081A06	Intron	+		No
		SALK_022324	Exon	+		No
AtSBT2.5	At2g19170	FLAG_181B06	Exon	+		No
AtSBT2.6	At4g30020	GK_125A08	Exon	+		No
		SALK_068944	Exon	+		No
AtSBT3.1	At4g21323	GK_069E04	Exon	+		No
AtSBT3.2	At1g32970	SALK_001743	1000 p	+		No
AtSBT3.3	At1g32960	SALK_086092	Exon	+		No
		SALK_107460	Exon	+		No
AtSBT3.4	At1g32950	SALK_040245	Exon	+		No
		SALK_058032	Intron	+		No
AtSBT3.5	At1g32940	GK_672C08	Intron	+		No
		SAIL_400_F09	Intron	+		No
AtSBT3.6	At4g10550	SALK_104806	Intron	+		Mild
AtSBT3.7	At4g10510	SALK_081645	Exon	+		No
AtSBT3.8	At4g10540	GK_226F04	Exon	+		No
AtSBT3.9	At4g10520	SALK_048279	Intron	+		No

AtSBT3.10	At4g10530	SALK_014429	Intron	+	No
AtSBT3.11	At5g11940	SALK_067858	Exon	+	No
AtSBT3.12	At4g21326	SALK_037231	Exon	+	Mild
AtSBT3.13	At4g21650	SALK_082160	Exon	+	No
AtSBT3.14	At4g21630	SALK_127987	Exon	+	No
AtSBT3.15	At4g21640	SALK_064593	Exon	+	No
AtSBT3.16	At1g66210	SALK_009433	Exon	+	Mild
		SALK_004741	Exon	+	Mild
AtSBT3.17	At1g66220	SALK_040473	Exon	+	No
		SALK_070765	Exon	+	No
AtSBT3.18	At4g26330	GK_360C11	Exon	+	No
AtSBT4.1	At2g39850	SALK_016756	Intron	+	No
		SALK_038521	Exon	+	No
AtSBT4.2	At4g15040	SALK_024853	Exon	+	No
AtSBT4.3	At5g59190	SALK_149055	Exon	+	No
		SALK_075909	Exon	+	No
AtSBT4.4	At5g59100	SALK_016547	1000 p	+	No
AtSBT4.5	At3g46840	GK_251C03	Exon	+	No
		SALK_078286	Intron	+	No
AtSBT4.6	At3g46850	SALK_091683	1000 p	+	No
AtSBT4.7	At5g58820	SALK_013603	Exon	+	No
AtSBT4.8	At5g58830	GT_5_112073	Exon	+	No
AtSBT4.9	At5g58840	SALK_060155	Exon	+	No
AtSBT4.10	At5g58810	SALK_119237	Exon	+	No
AtSBT4.11	At5g59130	GK_132H10	Exon	+	No
AtSBT4.12	At5g59090	GK_239B04	Exon	+	No
AtSBT4.13	At5g59120	SALK_009191	Intron	+	No
AtSBT4.14	At4g00230	SALK_019254	Exon	+	No
AtSBT4.15	At5g03620	SALK_063258	Exon	+	No
AtSBT5.1	At1g20150	SALK_017993	Exon	+	No
AtSBT5.2	At1g20160	SALK_012112	Exon	+	No
		SALK_012113	Exon	+	No
AtSBT5.3	At2g04160	SALK_051293	Intron	+	No
AtSBT5.4	At5g59810	GK_099F02	Exon	+	No
AtSBT5.5	At5g45640	SALK_107233	Exon	+	No
AtSBT5.6	At5g45650	GK_074B04	Intron	+	No
**AtSBT6.1**	**At5g19660**	**SALK_111474**	Exon	+	**Strong**
		**SALK_020530**	Exon	+	**Strong**
**AtSBT6.2**	**At4g20850**	**SALK_085776**	Exon	+	**Strong**

FST, flanking sequence tag; GK, GABI-Kat (http://www.gabi-kat.de/); SALK, http://signal.salk.edu/cgi-bin/homozygotes.cgi; UTR, untranslated region; 1000p: T-DNA insertion position 1000 bp upstream of promoter.

Table S3. *GLV1* transcript fold induction in transformed *sbt6* T-DNA mutant lines

Genotype	Transcript ratio relative to WT
WT	1.0
$GLV1^{OE}$	142.8
$GLV1^{OE}$ *sbt6.1-1* line 1[a]	192.4
$GLV1^{OE}$ *sbt6.1-1* line 2	4.7
$GLV1^{OE}$ *sbt6.1-1* line 3	20.8
$GLV1^{OE}$ *sbt6.1-1* line 4	11.6
$GLV1^{OE}$ *sbt6.1-1* line 5	22.9
$GLV1^{OE}$ *sbt6.1-1* line 6	95.2
$GLV1^{OE}$ *sbt6.1-1* line 7	24.7
$GLV1^{OE}$ *sbt6.1-1* line 8	2.6
$GLV1^{OE}$ *sbt6.2* line 1[a]	39.3
$GLV1^{OE}$ *sbt6.2* line 2	17.6

[a] Lines selected for further analysis.

Table S4. Propeptides and observed proteolytic products according to *m/z* for singly charged ions

Propeptide	Synthetic sequences*	Calculated *m/z*	Detected *m/z*	Cleaved peptides	Calculated *m/z*	Detected m/z
RALF23	SEIN**RRIL**ATRRYI	1761.0242	1761.4808	SEINRRIL	1000.5898	1000.8165
				ATRRYI	779.4522	779.6323
GLV1-1	NGGE**RRRAL**GGVE	1370.7247	1371.0605	NGGERRRAL	1028.5708	1028,7959
				NGGERRRA	915.4867	915.6780
GLV1-2	CASAA**RRLR**SHKHH	1629.8615	1630.7068	CASAARRLR	1003.5578	1003.9682
GLV1-3	ASAA**RRLR**SHKH	1389.7934	1390.0014	ASAARRLR	900.5486	900.6270

* Amino acids underlined correspond to reported subtilase canonical cleavage sites.

Table S5. Proteins identified after TAP purification with NTAP-Serpin1 expressed in *Arabidopsis* cell suspension cultures

Characteristics[a]	AT1G47710	AT5G19660
Description	Serpin1	SBT6.1
# Found/three experiments	03/mrt	02/mrt
Molecular mass (kDa)	42.7	116.7
Peptide count	16	17
Protein coverage (%)	41	16
Protein score	491	163
Protein score expectation value	2.60E-45	1.60E-12
Best Ions score	108	43
Best Ions score expectation value	2.20E-10	1.30E-03

Peptide mass spectrometry was done with the 4800 MALDI TOF/TOF[TM] Proteomics analyzer (AB SCIEX) and matching proteins were identified with the search engine Mascot version 2.1 (Matrix Science) with the TAIR8 database. Known TAP background proteins were filtered out.

[a] Peptide count, number of peptides with unique sequences matching the selected protein; Protein coverage %, percentage of protein sequence covered by assigned peptide matches; Protein score, score calculated by the Mascot search engine for each protein and based on the probability that peptide mass matches are nonrandom events, but if equal to or greater than the Mascot® Significance Level calculated for the database search, the protein match is considered to be statistically nonrandom at the 95% confidence interval. Protein score, -10*Log(*P*), where *P* is the probability that the observed match is a random event. Best Ions score, highest individual Ions Score for a given protein identification that is calculated by the Mascot search engine for each peptide matched from MS/MS peak lists and based on the probability that ion fragmentation matches are nonrandom events, but it is equal to or greater than the Mascot® Significance Level calculated for the database search, the peptide match is considered to be statistically nonrandom at the 95% confidence interval. Ions score, 10*Log(*P*), where *P* is the probability that the observed match is a random event.

Table S6. Typical subtilase target sequences in GLV peptides

Protein	Amino acid sequence
GLV1	MSCSLRSGL VIVFCFILLLLSSNVGCASAARRLRSHKHHHHKVASLDVFNGGERRRALGGVETGEEVVVMDYPQPHRKPPIHNEKS
GLV2	MAIRVSHKSFLVALLLILFISSPTQARSLREVVRNRTLLVVEKSQESRKIRHEGGGSDVDGLMDMDYNSANKKRPIHNR
GLV3	MMRFTIIVIAFLLIIQSLEEEHILVYAHEGGEAGHKSLDYQGDQDSSTLHPKELFDAPRKVRFGRTTRAEKEQVTAMNNDSWSFKISGEHKQTNILADHDTTKNTFCKKMMIIVNDLTSLPTLEPSTSTNDMEKLARLLRDDYPIYSKPRRKPPVNNRAPDKF
GLV4	MEMKKWSYANLITLALLFLFFIILLLAFQGGSRDDDHQHVHVAIRTKDISMGRKLKSLKPINPTKKNGFEYPDQGSHDVQEREVYVELRDYGQRKYKPPVHN
GLV6	MKLIRVTLFLCALAILLLVTPTSSLQLKHPYSSPSQGLSKKIVTKMATRKLMIISSEYVMTSTSHEGSSEQLRVTSSGKSKDEEKKLSEEEEEKKALAKYLSMDYRTFRRRRPVHNKALPLDP
GLV7	MTTLSKILCVLIILLLCFSFRYSLHEDGNQQSSRDFVSTAKAIKYGDVMKKMIRGRKLMMASGEKEEAETKMKRGNRETERNSSKSVEEDGLVAYTADYWRAKHHPPKNN
GLV8	MKKWSYAKLMTSALLLVFLSIILLAFHGGSRGDNHLYDHVAIGTKDILMGRKLKDLKPKTESLKMINPKKKNGFEYSDQVSSDLSRQEVFVDMMARDYQGPKPRSKPLKNN
GLV9	MKKTSLKLMTLVLGFCFVIYLLQGPRGGSRNGDLLIARKLISLEPIETKNAARSLKDSISTDLEEEVDRLMEHEYPSPVKPRKRTPVHNGVRNRH
GLV10	MSSIHVASMILLLFLFLHHSDSRHLDNVHITASRFSLVKDQNVVSSSTSKEPVKVSRFVPGPLKHHHRRPPLLFADYPKPSTRPPRHN
GLV11	MVSIRVICYLLVFSVLQVHAKVSNANFNSQAPQMKNSEGLGASNGTQIAKKHAEDVIENRKTLKHVNVKVEANEKNGLEIESKEMVKKRKNKKRLTKTESLTADYSNPGHHPPRHN

Typical subtilase target sequences are presented in red.
